# Seroprevalence of Coronavirus NL63 in Yamagata, Japan, Between 1976 and 2022

**DOI:** 10.1155/cjid/7854122

**Published:** 2026-05-18

**Authors:** Yohei Matoba, Naomi Ogawa, Tomoko Takahashi, Kenichi Komabayashi, Tatsuya Ikeda, Yoko Matsuzaki, Katsumi Mizuta

**Affiliations:** ^1^ Department of Microbiology, Yamagata Prefectural Institute of Public Health, Yamagata, 990-0031, Japan; ^2^ Division of Infection Control, Yamagata University Hospital, Yamagata, 990-9585, Japan

**Keywords:** acute respiratory infection, CoV-NL63, seasonal coronavirus, seroepidemiology, seroprevalence

## Abstract

Advances in the epidemiology of seasonal coronavirus (CoV) are important for understanding CoV transmission dynamics and strengthening the strategies against emerging CoV infections. The epidemiology of CoV‐NL63, a seasonal CoV discovered in 2003, is poorly understood, particularly before 2003. To analyze the seroprevalence of CoV‐NL63 in Yamagata, Japan, we performed a microneutralization test using 146 to 379 serum samples collected from four age groups ranging from 0 to 4 years to ≥ 40 years in 1976, 1990, 2010, 2019, and 2022, as well as the Yamagata isolate. We only measured the neutralizing antibodies (NT Abs) against CoV‐NL63, as methods for other seasonal CoVs had not yet been established. The total NT Ab‐positive rate in each year was 68.0%–90.4%. The Ab‐positive rates in 0–4‐year age group were 21.3%–65.2% in each year, whereas the rates for individuals aged above 4 years old were between 73.3% and 100% throughout the study period. We do not understand the influence of cross‐reactive immunity among seasonal CoVs. This study shows that seroprevalence against CoV‐NL63 increased with age in the younger generation and then plateaued at a high level, suggesting that reinfection may occur and that CoV‐NL63 was already transmitted among infants and children before CoV‐NL63 had been identified.

## 1. Introduction

Coronaviruses (CoVs) are enveloped RNA viruses that are broadly distributed among humans and other mammals, including bats [1]. CoVs have been arranged into four genera: alpha‐, beta‐, gamma‐, and delta‐CoVs, among which seven CoVs are known to be associated with human disease [1]. The four seasonal CoVs, the alphacoronaviruses CoV‐229E and CoV‐NL63, and the betacoronaviruses CoV‐OC43 and CoV‐HKU1 typically cause common colds, whereas severe acute respiratory syndrome CoV (SARS‐CoV), Middle East respiratory syndrome CoV (MERS‐CoV), and SARS‐CoV‐2 are highly pathogenic and often cause severe and/or fatal infections in humans [1, 2]. Among them, CoV‐229E and CoV‐OC43 were discovered in the 1960s, while the remaining CoVs were only identified after 2002 [1]. CoV‐NL63, the subject of this article, was first isolated and identified in 2003 from a 7‐month‐old child, who was admitted to the hospital with a diagnosis of bronchiolitis [3]. CoV‐NL63 has a worldwide distribution and infects both the upper and lower respiratory tracts, although most patients present with mild upper respiratory symptoms such as cough and rhinitis [4].

The Yamagata Prefectural Institute of Public Health (YPIPHEC) has carried out virus isolation among infants and children with acute respiratory infections since 1999 as part of its routine surveillance in collaboration with the National Epidemiological Surveillance of Infectious Diseases (NESID) in Japan [5]. We used a microplate method, including six cell lines (the HEF, Human Epithelial type 2 <HEp‐2>, VeroE6, MDCK, RD‐18S, and GMK cell lines). The seasonal CoVs were not isolated by the above cell lines, although 229E was later found to be isolated by the RD‐18S cell line [6]. Thus, the molecular method was actively introduced to allow detection of CoV genomes, and the findings in Yamagata between 2010 and 2019 revealed that winter was the major season for the four seasonal CoV infections among children [2]. An air‐liquid interface (ALI) culture using primary human airway epithelium enabled us to isolate all four seasonal CoVs and CoV‐NL63, in particular, could be subcultured using the LLC‐MK2 cell line after isolation using ALI culture [7]. Previous seroepidemiological studies of CoV‐NL63 were performed with immunoassays such as an enzyme‐linked immunosorbent assay (ELISA) [8–12] without using live viruses, except for one study [13]. Analyses were rarely conducted for the period before the identification of CoV‐NL63 in 2003 [12]. Knowledge of the historical seasonal CoV epidemiology might be important for understanding the CoV transmission dynamics, and it might also be helpful in strengthening the strategies against emerging CoV infections such as SARS‐CoV‐2. Thus, in this study, a longitudinal seroepidemiological study was conducted to clarify the incidence of CoV‐NL63 infection in Yamagata before and after the discovery of CoV‐NL63.

## 2. Materials and Methods

Human serum samples were collected for the National Epidemiological Surveillance of Vaccine‐Preventable Diseases led by the Ministry of Health, Labour and Welfare, Japan, under the Preventive Vaccination Law. Serum samples were collected from residents in Yamagata between May and August, when the influenza virus was not circulating. Informed consent was obtained (either from the individual or guardian) from 2000 onwards, whereas samples were collected without informed consent before 2000, when informed consent was not yet strictly required. In this study, serum samples collected in 1976, 1990, 2010, 2019, and 2022 were used.

Neutralizing antibody (NT Ab) titers against the CoV‐NL63 Yamagata isolate (NL63/Yamagata.JPN/2016‐854; GenBank accession number LC895504) were measured using a microneutralization test similar to those used in previous studies [14]. NL63/Yamagata.JPN/2016‐854 was isolated using ALI culture and was further subcultured and propagated in the LLC‐MK2 cell line [7]. Serum samples were serially diluted two‐fold from 1:8 to 1:512 and the LLC‐MK2 cell line was used. The reciprocal value of the highest dilution of serum resulting in no or only a weak cytopathic effect compared with the control was taken to be the titer. Seropositivity was defined as an NT Ab titer ≥ 1:8, consistent with our previous study [14] and comparable to the 1:10 used in another study [13].

This study was conducted with approval from the ethics committee of the YPIPHEC 25‐07.

## 3. Results

The number of serum samples collected in 1976, 1990, 2010, 2019, and 2022 and the rate of Ab‐positive cases for each year in each age group (0–4 years, 5–19 years, 20–39 years, and ≥ 40 years) for this study are shown in Table 1. The Ab‐positive rate for the total serum samples in each year was 68.0%–90.4%. The Ab‐positive rates in each year for the 0–4‐years age group were 21.3%–65.2%, whereas Ab‐positive rates in individuals aged above 4 years old were 73.3%–100%.

**TABLE 1:** Number of serum specimens collected and neutralizing antibody‐positive rates (%) for the seroepidemiological study of CoV‐NL63 by age group between 1976 and 2022 in Yamagata, Japan.

Age (years)	1976		1990		2010		2019		2022	
	No.	Positive^a^ rate (%)	No.	Positive rate (%)	No.	Positive rate (%)	No.	Positive rate (%)	No.	Positive rate (%)
0–4	38	31.6	17	58.8	47	21.3	37	64.9	23	65.2
5–19	51	84.3	48	87.5	71	93.0	41	85.4	47	93.6
20–39	45	73.3	32	100	146	93.2	57	84.2	101	92.1
≥ 40	44	75.0	49	98.0	115	90.4	67	86.6	57	94.7
Total	178	68.0	146	90.4	379	83.4	202	81.7	228	90.4

^a^Serotiter ≥ 1:8.

**FIGURE 1:**
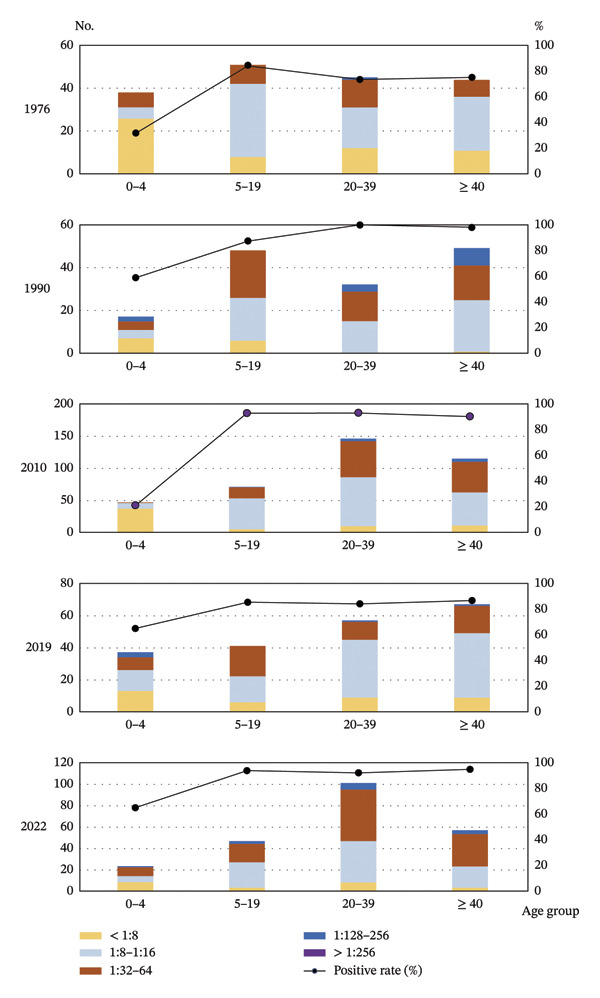
Neutralizing antibody titers and seroprevalence of CoV‐NL63 among different age groups in Yamagata, Japan, between 1976 and 2022. The bar and the left *y*‐axis show the number of individuals in each age group, and the line and the right *y*‐axis show the seroprevalence (%).

The numbers of Ab‐positive cases with different Ab titers for each year in each age group are shown in Figure 1. The rates of Ab‐positive cases for each year in each age group are also shown in Figure 1. The ratio of serotiters 1:8–1:16 and 1:32–1:64 accounted for 43.2%–68.6% and 30.6%–50.0%, respectively, of seropositive individuals in each year, with those of 1:128–1:256 accounting for 0.8%–9.8%. The number of residents who had a serotiter > 1:256 was only one in 2019.

## 4. Discussion

The previous study in Yamagata and other studies suggested that CoV‐NL63 infections are common during childhood and seropositivity against CoV‐NL63 increases with age. The previous Yamagata study showed that CoV‐NL63 infections occur almost every year, mainly in the winter season, among infants and children under 6 years old with a median age of 3 years [2]. Hofmann et al. reported that infants between 3 and 6 months of age did not contain NT Abs against the spike glycoprotein of CoV‐NL63, whereas all sera from adults contained NT Abs at high levels [8]. A seroepidemiological study using an ELISA indicated that children have a high level of Abs against CoV‐NL63 at birth as maternal Abs, and the level of these Abs decreased to very low levels within 3 months [9]. Thereafter, the numbers of seropositive individuals increased with age: 29%–33% between 6 months and 1.5 years; 75% at 2.5–3.5 years; and the majority of children aged over 3.5 years were seropositive [9]. Shao et al. reported that the percentage of seropositive individuals against the nucleocapsid protein of CoV‐NL63 was 45.2% for infants < 2 months old, 4.7%–11.1% for infants 2–5 months old, and 25.0%–70.3% for individuals aged over 6 months [10]. Sayama et al. reported that the seroprevalence of CoV‐NL63 showed 90% positivity by 3–4 years of age [11]. The findings in this study in Yamagata showed that the seroprevalence against CoV‐NL63 increased with age in the younger generation and then plateaued at over 73.3% among individuals aged above 4 years old throughout the study period. This again strongly supports the notion that CoV‐NL63 infections occur among young children.

Another finding is that high seropositive rates persist in individuals aged 5 years and older, possibly indicating that reinfections occur frequently after initial CoV‐NL63 infection by age 5. Ab titers acquired after CoV infection can decline, as shown by Edridge et al. as follows: 0‐12 times, infections of four seasonal CoVs were detected by the ELISA method among 10 adults during a follow‐up period of 138–342 months since 1985, and protective immunity was only short‐lived [12]. Thus, we might be affected by these viruses repeatedly during our lives.

Lynch et al. reported that NT Abs were detected in 71% of the plasma samples in healthy adults in Australia in 2020 [13], and the 73.3%–100% observed in this study is slightly higher. Although they used CoV‐NL63 (Amsterdam‐1 strain) and a cut‐off titer of 1:10, we remain unsure why the seroprevalence was higher in Yamagata than in Australia. We reported that CoV‐NL63 circulated endemically among children between 2010 and 2019, with peaks occurring almost every winter in Yamagata [2]. Thus, the seroprevalence observed in this study in 2010 and 2019 reflects data obtained under such endemic conditions, using our method.

We are not sure whether there are cross‐reactive Abs among seasonal CoV infections. The interpretation of cross‐immunity among CoVs is reportedly difficult, and the degree of cross‐immunity remains unclear, as the full histories of seasonal CoV exposures are unknown [15–17].

As CoV‐OC43 and CoV‐HKU1 can only be grown in ALI culture [7] and the methods for measuring NT Abs against CoV‐229E with the RD‐18S and/or Hela‐ACE2‐TMPRSS2 cell lines [6, 18] are not yet established in our laboratory, we did not measure the NT Abs against other seasonal CoVs in this study.

The tendencies in Ab positivity rates by age group in the previous studies described above were also observed in this study when Abs from 1976 and 1990 were used, prior to the discovery of CoV‐NL63 in 2003. Therefore, this seroepidemiological study in Yamagata indicates that the transmission of CoV‐NL63 has continued at least since the 1970s. CoVs have been shown to have spilled over from zoonotic reservoirs, such as bats, through intermediate animal hosts [1, 19]. A phylogenetic analysis using CoVs from wild animals as well as humans indicated that CoV‐NL63 diverged from CoV‐229E in around 1927 and has infected humans since then [19]. CoV‐NL63 was detected in human samples collected in 1981 and 1988 in a retrospective investigation [20, 21]. This study in Yamagata does not contradict these results and conclusions.

The findings of this Yamagata study are subject to limitations. The serum specimens were old, and we are not sure how much the NT Ab titers may have decreased during storage. However, the previous Yamagata studies showed that NT Ab titers up to at least 1:1024 could be measured [14].

## 5. Conclusions

This longitudinal seroepidemiological study in Yamagata suggested that CoV‐NL63 had already been in circulation between the 1970s and 1990s, before its identification.

## Author Contributions

Tatsuya Ikeda, Yoko Matsuzaki, and Katsumi Mizuta conceived of the present study. Yohei Matoba, Naomi Ogawa, Tomoko Takahashi, Kenichi Komabayashi, and Katsumi Mizuta performed virus culture and measured the NT Abs.

## Funding

The authors received no specific funding for this work.

## Disclosure

All authors were involved in the critical appraisal of the manuscript and gave final approval of the version to be published.

## Ethics Statement

This study was conducted with approval from the ethics committee of the Yamagata Prefectural Institute of Public Health (YPIPHEC 25‐07).

## Consent

Human serum samples were collected for the National Epidemiological Surveillance of Vaccine‐Preventable Diseases led by the Ministry of Health, Labour and Welfare, Japan, under the Preventive Vaccination Law. Serum samples were collected from residents in Yamagata. Informed consent was obtained (either from the individual or guardian) from 2000 onwards, whereas samples were collected without informed consent before 2000, when informed consent was not yet strictly required.

## Conflicts of Interest

The authors declare no conflicts of interest.

## Data Availability

The data that support the findings of this study are available from the corresponding author upon reasonable request.
